# New determinants for casual peripheral mechanism of neurogenic lung edema in subarachnoid hemorrhage due to ischemic degeneration of vagal nerve, kidney and lung circuitry. Experimental study^1^

**DOI:** 10.1590/s0102-865020190030000003

**Published:** 2019-03-18

**Authors:** Celaleddin Soyalp, Mehmet Nuri Kocak, Ali Ahiskalioglu, Mehmet Aksoy, Canan Atalay, Mehmet Dumlu Aydin, Murteza Cakir, Cagatay Calikoglu, Sevilay Ozmen

**Affiliations:** I MD, Department of Anesthesiology, Medical Faculty, Yil University, Van, Turkey. Conception and design of the study, acquisition of data, manuscript writing.; II MD, Department of Neurology, Medical Faculty, Ataturk University, Erzurum, Turkey. Technical procedures, manuscript preparation.; III Assistant Prof., Department of Anesthesiology and Reanimation, Medical Faculty, Ataturk University, Erzurum, Turkey. Acquisition, analysis and interpretation of data; manuscript preparation; critical revision.; IV Prof., Department of Neurosurgery, Medical Faculty, Ataturk University, Erzurum, Turkey. Histopathological examinations, manuscript writing, critical revision, final approval.; V MD, Department of Pathology, Medical Faculty, Ataturk University, Erzurum, Turkey. Histopathological examinations.

**Keywords:** Subarachnoid Hemorrhage, Renal Artery, Lung, Edema, Rabbits

## Abstract

**Purpose:**

To evaluate whether there is a relationship between renal artery vasospasm related low glomerular density or degeneration and neurogenic lung edema (NLE) following subarachnoid hemorrhage.

**Methods:**

This study was conducted on 26 rabbits. A control group was formed of five animals, a SHAM group of 5 to which saline and a study group (n=16) injected with homologous blood into the sylvian cisterna. Numbers of degenerated axons of renal branches of vagal nerves, atrophic glomerulus numbers and NLE scores were recorded.

**Results:**

Important vagal degeneration, severe renal artery vasospasm, intrarenal hemorrhage and glomerular atrophy observed in high score NLE detected animals. The mean degenerated axon density of vagal nerves (n/mm^2^), atrophic glomerulus density (n/mm^3^) and NLE scores of control, SHAM and study groups were estimated as 2.40±1.82, 2.20±1.30, 1.80±1.10, 8.00±2.24, 8.80±2.39, 4.40±1.14 and 154.38±13.61, 34.69±2.68 and 12.19±1.97 consecutively. Degenerated vagal axon, atrophic glomerulus and NLE scores are higher in study group than other groups and the differences are statistically meaningful (p<0.001).

**Conclusion:**

Vagal complex degeneration based glomerular atrophy have important roles on NLE following SAH which has not been extensively mentioned in the literature.

## Introduction

 Neurogenic lung edema (NLE) can be originated from vagosympathetic imbalance^1^ following SAH. Vagal insufficiency and hypo-functioned hilar parasympathetic ganglia may be a causative role on the pathogenesis of NLE^2^. Vagal ischemia and cervical spinal network degenerations trigger respiratory disturbances because vagal ischemia also cause NLE by destroying heart functions. Cerebral fat embolism induced by SAH^3^ could cause worsened prognosis of NLE because NLE has an important roles on systemic chylomicron embolism. SAH induced vagal lesions cause acute kidney injury^4^. SAH is prevalent causes of morbidity and mortality among dialysis patients. And even, chronic kidney disease is associated with higher stroke and SAH incidence^5^. Sodium retention related fulminant nephrotic edema arising from sympathetic over activity may aggravate NLE. Vagal micro-ganglia and plexus insufficiency around renal tissue^6^ or increased sympathetic outflow cause renovascular hypertension^7^. Because the vagal stimulation prevents renal inflammation and renovascular pathologies. Interruption of vagal impulses inhibit hyperactive sympathetic receptors of kidneys^8^ and renal sympathetic activity increase in vagotomised rats^9^. Although renal vasospasm is a dangerous complication of subarachnoid hemorrhage, nobody mentioned vagal insufficiency based neurogenic lung edema (NLE). The aim of this study was to elucidate whether there is a relationship between vagal ischemia induced renal artery vasospasm triggered glomerular degeneration and NLE following subarachnoid hemorrhage. 

## Methods 

 Animal husbandry and the study design followed the guidelines of the National Institutes of Health. The study plan was approved by the Ethic Committee of Ataturk University. All experimental protocols were conducted Pharmacology Laboratory, Ataturk University, School of Medicine, Erzurum.

This experimental study was conducted on 26 rabbits. Five rabbits (n=5) used to analyze the normal structure of the vagal nerve roots, lungs and kidney tissues. The remaining’s (n=21) anaesthetized by subcutaneous injection of a mixture of ketamine hydrochloride (25 mg/kg), lidocain hydrochloride (15 mg/kg), and acepromasine (1mg/kg). After preparing the occipital region, five rabbits of (n=5) received an 1cc saline injection into the sylvian cisterna for SHAM group (n=5). SAH was produced by the injection of 0.5cc blood into sylvian cisterna taken from auricular arteries (n=16) once a day/three days periods. Animals followed-up two weeks then were sacrified. Their lungs, vagal nerve roots and kidneys were removed and preserved in 10% formalin solution for seven days. The specimens embedded in paraffin blocks and consecutive twenty sections of 5 µm were taken for the stereological examinations. Vagal nerve roots, kidney and lung tissues were stained with hematoxylene and eosin (H&E) and tunnel methods. All preparations were analysed by Stereological and cavaliery methods and data analysed by statistical methods. Histopathologically, cytoplasmic condensation, nuclear shrinking, cellular angulations and peri-cytoplasmic halo formation secondary to cytoplasmic regression and Tunnel staining positivity were considered as the criteria of epithelial degeneration in kidneys and lungs.

Physical dissector method was used to evaluate the numbers atrophic glomerulus numbers. Two dissector pairs were mounted on each slide. A counting frame was placed on consecutive section photographs on screen of PC for counting of neurons. The bottom and the left hand edges of the frame were excluded for exclusion lines together with the extension lines. Other boundaries of the frame and the top-right corner were considered to be inclusion points and any particle which hit these lines or was located inside the frame counted as a dissector particle. Glomerules of kidneys were counted if they were visible in the reference section. Reference and look-up sections were reversed in order to double the number of dissector pairs without taking new sections. The average numerical density of glomerules (NvGN) per mm3 was estimated using the following formula: NvGN=ΣQN/txA

Where ΣQN is the total number of counted glomerules appearing only in the reference sections; t is the section thickness and A is the area of the counting frame. Cavalieri volume estimation method was used to obtain the total number of glomerules in each specimens. Total number of neurons was calculated by multiplication of the volume (mm^3^) and numerical density of glomerules in each kidney were counted by stereological methods. 

NLE scores were classified as: Normal-0, minimally edema-1, pulmonary artery vasospasm-2, alveolar hemorrhage-3, lymph node enlargement-4 and alveolar rupture-5, and that scores evaluated between 0-15. NLE scoring system is designed by ourselves according to clinical and neuropathological findings of neurogenic edema. Namely that scoring system was arranged by simple to complex histopathological findings of NLE^3^.

To estimate atrophic glomerulus numbers, one hundred section was taken from each kidneys and normal/atrophic glomerulus numbers were estimated by Stereological methods. The relationship between the degenerated vagal axon, atrophic glomerulus and NLE scores were analyzed statistically. Statistical analysis was performed on IBM SPSS 20.0 (SPSS Inc., Chicago, Illinois, USA) software. The distribution of variables was evaluated for normality using the Shapiro-Wilk tests. Descriptive data were expressed as mean ± standard deviation. Normally, distributed data comprising continuous variables were analyzed using ANOVA test. Otherwise, the Kruskal-Wallis test was used. P < 0.05 was considered statistically significant.

## Results 

 Two of 15 rabbits died within the second week, likely due to cardiorespiratory irregularities and new animals were restudied. Four animals in the study group died. In control group, the heart rate was 252±31 beats/min, the respiration rate was 24±4 breaths/min and the arterial oxygen saturation was 96±4%. Early phase of SAH, the heart rate decreased to 156±12 beats/min, the breathing rate was 13±3 breaths/min, and the oxygen saturation was 83±10%. Late phase of SAH, the pulse rate increased to 349±31 beats/min, while the respiration rate increased to 41±11 breaths/min with severe tachypnea and apneic variabilities. ST depression, ventricular extrasystols, bigeminal pulses, QRS separation, and fibrillations were observed in animals with SAH. Finally, decreased respiration amplitude, shortening of inspiration with prolonged expiration time, apnea-tachypnea attack, diaphragmatic breath and respiratory arrest observed in dead animals. Massive neurogenic lung edema detected in important glomerulus degeneration developed animals.

**Figure 1:**
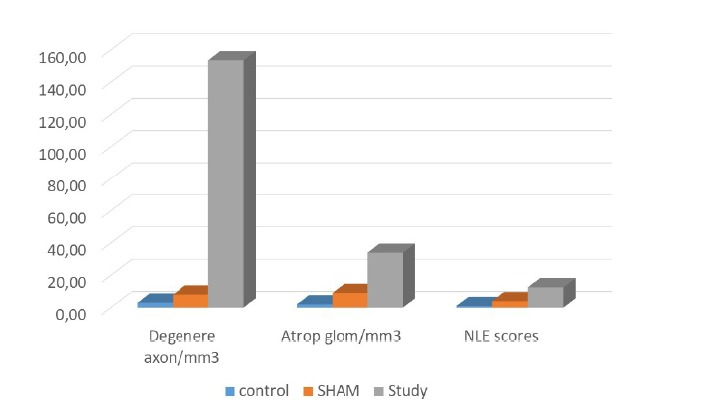
Numerical results of study.

Important degenerative changes detected in vagal axons in severe hypertension detected animals. Severe renal artery vasospasm, intrarenal hemorrhage and renal glomerular degenerations observed in hypertensive rabbits. Normal blood pressure was estimated as 98±10/mmHg in normal rabbits, 105±12/mmHg in SHAM, 112±14/mmHg in middle (n=10), and >122±10/mmHg in severe glomerular degeneration detected animals (n=6). The mean degenerated axon density of vagal nerves (n/mm^2^), atrophic glomerulus density (n/mm^3^) and NLE scores of control, SHAM and study groups were estimated as 2.40 ±1.82, 2.20±1.30, 1.80±1.10; 8.00±2,24 8.80±2.39, 4.40±1.14 and 154.38±13.61, 34.69±2.68 and 12.19±1.97 consecutively. Statistical analysis between the axonal degeneration, low density or atrophic glomerulus numbers and NLE scores were not meaningful in SHAM (p>0.05), but meaningful in study group (p<0.001). Numerical values are summarized in Table 1 and Figure 1.

**Table 1:** Numerical results of study.

	Group A	Group B	Group C
Degenerated axon density of vagal nerves (n/mm^2^)	3 (0-4)	8 (5-11)	153.50 (134-188)^α, β^
Atrophic glomerulus density (mm^3^)	2 (1-4)	9 (6-12)	34 (32-42)^α, β^
NLE scores	1 (1-3)	4 (3-6)	12.50 (9-15) ^γ, β^

All values given median (minimum-maximum), Group A: Control Group, Group B: SHAM group, Group C: Study group, NLE: Neurogenic lung edema.

^α^ *p* < 0.001 between Group C and A, ^β^ *p* < 0.001 between Group C and B, ^γ^ *p* < 0.001 between Group C and A

**Figure 2:**
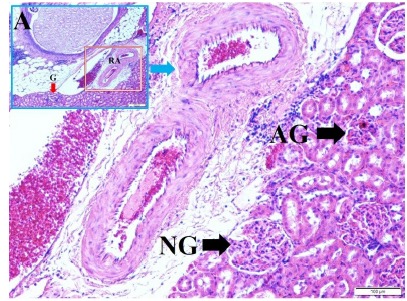
Histopathological appearance of renal arteries (**RA**) and glomerulus of kidney (LM, H&E, x4/A); normal (**NG**) and atrophic glomerulus is seen in SAH created rabbit (LM, H&E, 10).

**Figure 3:**
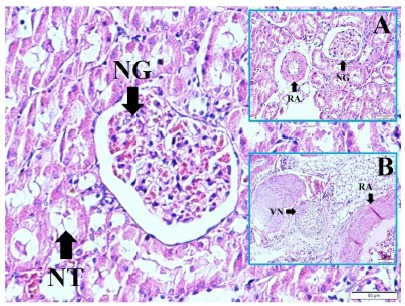
Histological representation of paranchymal renal arteries (**RA**) and normal glomerulus (**NG**) (LM, M&E, x4/A), renal branches of vagal nerves (**VN**) near the renal arteries (**RA**) (LM, H&E, x4/B) and normal tubulus (**NT**) with normal glomerulus (**NG**) is seen in a normal rabbit (LM, H&E, x20/Base).

Histopathological findings were summarized as follows. Figure 2 shows histopathological appearance of renal arteries and glomerulus of kidneys in normal and atrophic glomerulus in SAH created rabbit. Histological representation of paranchymal renal arteries and normal glomerulus, renal branches of vagal nerves near the renal arteries and normal tubulus with normal glomerulus is seen in a normal rabbit in Figure 3. 

**Figure 4:**
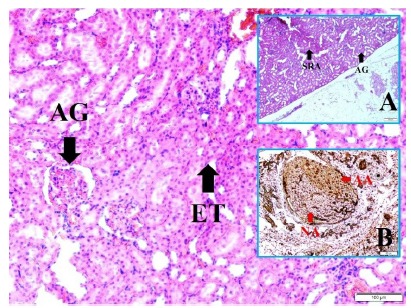
Axon numbers estimation method is illustrated in renal branches of vagal nerves. Degenerated and normal axons were counted in per ¼ of all quadrants and multiplied by 4 (LM, H&E, x10/B).

**Figure 5:**
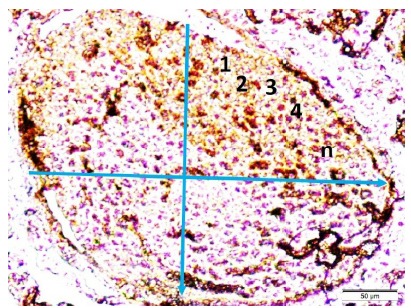
Glomerulus numbers estimation method is illustrated in renal tissues. Degenerated and normal glomeruluses were counted in per mm^3^ of all consequtive four histological sections cutted with 5 micrometers silices (LM, H&E, x10/4).

**Figure 6:**
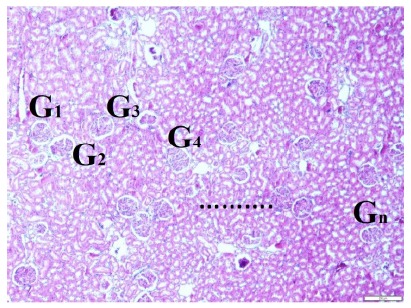
Histopathological appearances of NLE developed sample with spasm detected pulmonary artery (**SPA**), alveolar rupture (**R**) (LM, H&E, x10/Base), hemorrhage (**H**) (LM, H&E, x4/A) and enlarged lymph node (**LN**) (LM, H&E, x4/B) of a SAH created animal.

Figure 4 shows histological representation of paranchymal spastic renal arteries and atrophic glomerulus, renal branches of vagal nerves near the renal arteries with normal and degenerated axons and atrophic glomerulus with edematous tubules in a SAH created rabbit. Axon numbers estimation method is illustrated in renal branches of vagal nerves. Degenerated and normal axons were counted in per ¼ of all quadrants and multiplied by 4 (Fig. 5). Figure 6 shows glomerulus numbers estimation method is illustrated in renal tissues. Degenerated and normal glomeruluses were counted in per mm^3^ of all consecutive four histological sections cutted with 5 micrometers. Histopathological appearances of NLE developed sample with spasm detected pulmonary artery, alveolar rupture, hemorrhage and enlarged lymph node of a SAH created animal (Fig. 6). 

## Discussion

### A synopsis of study

 Autonomic control of the lungs are maintained by cervical sympathetic-vagal network and cervicothoracic spinal nerves. Neurogenic lung edema (NLE) is known as a serious complication of SAH, but the rational neuronal mechanism of that over activity has not yet been understood. High neuron density of stellate ganglion may have important roots in sympathetic over activity-related NLE in SAH^1^. Cervical dorsal root ganglion degenerations result in cardiorespiratory disturbances^10^. Vagal ischemia may be a causative role on the pathogenesis of NLE. Degenerated vagal complex may be a causative factor in the lymph node infarct in SAH^11^. Failure hilar parasympathetic ganglia may be responsible for pulmonary vasospasm in lungs. SAH related NLE cause systemic chylomicron embolism^3^ in kidneys and rely on renal insufficiency. Vagal ischemia originated renal vagal network degeneration increase in renal sympathetic over activity focused renal vasospasm /renal hypertension/glomerular injury/renal insufficiency induced NLE. 

### Vagus and renal innervation

 Renal tissues are innervated by vagal nerves, sympathetic trunks and thoracolumbar somatosensitive fibers and renal functions are regulated by that neural network^12^. Vagal efferent and afferent preganglionic neurons are seen in the suprarenal ganglia. These vagal contacts may directly control the postganglionic outflow and modulate postganglionic vagal inputs which exert sympathetic outflow^13^. Vagal efferents terminated in micro ganglia and periarterial sympathetic named as renal plexuses and maintain the essentials roles of kidney. Parasympathetic innervation deficiency of the adrenal glands^6^ should be considered as a factor for classical renovascular hypertension. Baroreflex dysfunction cause in renal failure by aortic vagal efferent nerve ischemia^14^. Cardiopulmonary reflexes and body fluid electrolyte imbalances controlling could not be regulated by vago-sympathetic network following SAH^15^. 

Increased sympathetic activity and reduced cardiac vagal tone aggravate hypertension, kidney disease, heart failure^7^. Efferent renal sympathetic nerve activity increase in vagotomised rats^9^. Impaired reflex control of heart rate is frequently disrupt via depressed baroreflex sensitivity following myocardial infarction^16^. Under appropriate conditions vagal afferents stimulate renal release of dopamine and produce a neurogenically mediated natriuresis^17^.

Cervical vagotomy abolished the hemorrhage-induced inferior cardiac sympathetic activity and renovascular hypertension^18^. Interruption of the impulse by vagotomy result of inhibit suppression of the hyperactive sympathetic receptors on kidneys^8^. Prolonged ischemia during reperfusion are mediated by activation of vagal and sympathetic afferent endings on kidneys^19^. 

Vagotomy alters sympathetic outflow predominantly to the renal circulations. Sympathetic control of the kidney and spleen can be selectively maintained by sympathetic outflow^20^. Renal and adrenal sympathetic nerve activity cause acute renal hemorrhage secondary to inputs decreased vagal activity^21^. Activation of cardiac sympathetic afferent neurons can lead to alterations in excretion of water and electrolytes^22^. 

Baroreflex neural regulation of renal blood flow controls carotid sinus and vagus nerves^23^. Ischemia of carotid sinus baroreceptors contribute to deteriorated baroreceptor reflexes^24^. If carotid baroreflex arc lesioned; arterial pressure, heart rate and renal vascular conductance deteriorated^25^. Systemic hypoxia markedly potentiates the reflex renal constriction^26^. In our model, vagal ischemia based renovascular hypertension are discussed.

### Subarachnoid hemorrhage renal disease relations

 Intracerebral hemorrhage, cerebral infarction, and SAH remain a dangerous causes of morbidity and mortality among dialysis patients. SAH increases the incidence of acute kidney injury^4^. Acute kidney injury is common in critically ill patients and may contribute to poor outcome and renal functions can be disordered in SAH. Kidney transplantation recipients have a higher risk of SAH compared to the general population^27^. Isolated anterior spinal artery aneurysms may cause renal dysfunction in SAH patients^28^. Chronic kidney disease is associated with higher stroke and SAH incidence^5^. Hypertonic saline therapy is a risk factors for developing acute kidney injury following SAH^29^. Hyponatremia is common complication in neurosurgical patients and associated with significant morbidity and mortality. In our opinion, glomerulopathies may be an important factor in such cases. Hypokalemia is a common electrolyte disorder in the intensive care unit. Hyponatremia is often associated with, and worsens, the prognosis of severe aneurysmal SAH. Several sodium and water intake have been described in SAH^30^. Hyponatremia may be originated from SAH induced glomerular disease. Hyponatremia is frequently seen in neurosurgical patients and generally attributed to inappropriate secretion of antidiuretic hormone. According to this knowledge; we announced that disordered sodium metabolism should be regarded as renal diseases related NLE.

Based on our findings, we hypothesized that vagal ischemia induced by SAH rely on descendant degeneration renal vagal network and developed renal sympathetic overactivity and renal vasospasm cause renal hypertension, glomerular injury and renal insufficiency presented by body fluid collection and also NLE which has not been mentioned in the literature.

### Limitations

 If renal angiography, ultrasonography or other invasive procedures and biochemical analysis should be done the better results could be obtained.

The animals were divided into three group as five (n=5) for the control group, five for the SHAM group (n=5) and the remaining sixteen (n=16) for the study group. Many researchers consider five animals per group as adequate sample size, but after reviewing recently published papers of on this issue, it can come to the conclusion that this notion of five animals per group has a little scientific and statistical basis. 

## Conclusion

 Vagal complex degeneration based glomerular atrophy have important roles on NLE following SAH. 
